# Trichlorophenate of Lime

**Published:** 1883-09

**Authors:** 


### Trichlorophenate of Lime.

A Russian medical journal publishes an account of experiments made by one of its collaborators on the antiseptic properties of trichlorophenate of lime, a mixture of phenic acid and chloride of calcium. From it, it appears that this compound is twenty times stronger as an antiseptic than phenic acid, permanganate of potassa, chloride of calcium, salicylic acid, or boric acid. It is not only disinfectant, but it removes all odors from wounds. It is essentially useful in soft chancre, diptheria, and other gangrenous affections. Trichlorate of phenol is not only easily and quickly prepared, but its price is also less than that of phenic acid.—*Medical Press.*

